# Factors Influencing the Age of Complementary Feeding—A Cross-Sectional Study from Two European Countries

**DOI:** 10.3390/ijerph16203799

**Published:** 2019-10-09

**Authors:** Monika A. Zielinska, Petra Rust, Daria Masztalerz-Kozubek, Jacqueline Bichler, Jadwiga Hamułka

**Affiliations:** 1 Department of Human Nutrition, Faculty of Human Nutrition and Consumer Sciences, Warsaw University of Life Sciences—SGGW, 02-776 Warsaw, Poland; monika_zielinska@sggw.pl (M.A.Z.); daria.masztalerz@gmail.com (D.M.-K.); jadwiga_hamulka@sggw.pl (J.H.); 2 Department of Nutritional Sciences, University of Vienna, 1090 Vienna, Austria; jacqueline.bichler@gmx.net

**Keywords:** breastfeeding, introduction of complementary feeding, infant feeding, introduction to solids, preterm infants, risk factors, weaning

## Abstract

The timing of introducing complementary feeding affects nutritional status and children’s health. The aim of this study was to determine sociodemographic and birth-related factors associated with the age of introducing complementary foods. This cross-sectional study investigated parents (*n* = 5815) of children aged 12–36 months from Poland (*n* = 4065) and Austria (*n* = 1750) using a single online questionnaire. During the study, detailed data about sociodemographic characteristics, variables related to pregnancy, and early feeding practices were collected. Univariate and multivariate logistic regression models were used to investigate factors associated with the introduction of complementary feeding before 4 completed months, between 4 and 6 months, and after 6 completed months separately for both countries. Complementary foods were introduced before 4 months in 3.0% of infants (2.4% in Poland and 4.3% in Austria), between 4 and 6 months in 65.0% (60.5% in Poland and 75.3% in Austria), and after 6 completed months in 32.1% of infants (37.1% in Poland and 20.4% in Austria). The factors related to earlier introduction of complementary feeding were lower maternal age (in Austria 25–29 years: aOR 2.21 (95% CI 1.06–4.65)) and education level (in Poland and Austria primary and vocational: aOR 14.49 (95% CI 3.73–56.35), aOR 2.13 (95% CI 1.10–4.11), respectively), preterm birth (in Poland and Austria: aOR 10.21 (95% CI 5.73–18.20); aOR 4.45 (95% CI 2.42–8.18), respectively), never breastfeeding (Poland: aOR 2.73 (95% CI 1.29 – 5.76)) and receiving an infant formula after hospital discharge (in both countries: aOR 3.73 (95% CI 2.06–6.75); aOR 3.65 (95% CI 1.87–7.12), respectively). These factors should be taken into account by health professionals in identifying mothers who are least likely to follow nutritional recommendations.

## 1. Introduction

The first two years of children’s lives are a time of rapid growth and development. Proper nutrition during this period is crucial for achieving the optimal development and good health in further life [1]. Exclusive breastfeeding for the first six months of infant life is recognized as a gold standard in infant nutrition associated with better health outcomes both in children and mothers [2]. During this time, breastmilk provides sufficient energy, macronutrients, and most of the micronutrients to meet the requirements of healthy term-born infants [3]. Later on, breastmilk alone may no longer be sufficient to meet the nutritional requirements of infants, especially for iron, zinc, vitamin A, D, E, protein, and energy [3,4,5]. At this time, the complementary feeding period begins, when solids are progressively introduced, and extended up to the end of the second year of life, ideally along with a continuation of breastfeeding [1,4]. It is an important period from a nutritional, developmental, as well as social perspective. Complementary foods provide essential nutrients, but complementary feeding is also an important stage in the transition from milk feeding to family foods [6]. However, the introduction of complementary feeding requires an optimal level of physiological and neurological maturation for it to be successful [6,7].

The World Health Organization (WHO) recommends introducing complementary feeding after 6 months with continued breastfeeding up to two years and beyond [8,9]. The European Society for Paediatric Gastroenterology, Hepatology, and Nutrition (ESPGHAN), as well as European Food Safety Agency (EFSA) recommend that complementary foods should be introduced between 4 and 6 months but should not be delayed beyond 6 months [6,10]. However, the ESPGHAN endorses exclusive or predominant breastfeeding for approximately 6 months as a goal in infant nutrition [6]. Polish, as well as Austrian recommendations, are consistent with European recommendations—complementary feeding between 17 and 26 weeks of infant life is recommended; however, 6 months of exclusive breastfeeding are recommended [11,12].

The proper timing of the introduction of complementary feeding is crucial due to its health outcomes. Previous studies have been shown that too early complementary feeding (<4 months) may be associated with an increased risk of childhood obesity [13], but an Australian cohort has demonstrated that the duration of breastfeeding may be more important than the timing of solids introduction [14]. Data concerning the time of complementary feeding introduction and risk of allergy are also ambiguous [15]. Despite the lack of strong evidence that early introduction to solids (before 6 months) can be associated with negative health effects, this is a factor that indirectly affects the cessation of exclusive breastfeeding. A shorter time of exclusive breastfeeding reduces the health benefits for both mother and child [2]. In preterm infants, a higher rate of hospital admissions was observed in infants who had been introduced to solids at the 4 months of corrected age compared to the 6 months group. However, the authors did not observe any differences in weight-for-age z-score in the twelfth month [16].

Data from nearly 80 countries around the world have shown that 5%, 11%, and 29% of infants aged 0–1, 2–3 and 4–5 months old, respectively, had already been introduced to solids. In Central and Eastern Europe and the Commonwealth of Independent States regions, these percentages were lower—1%, 5%, and 22%, respectively [17]. A study from five European countries (Germany, Belgium, Italy, Poland, and Spain) observed that 37% or 6% of formula-fed and 17% or 0.6% of breastfed infants already received solids before the age of 4 or 3 completed months despite the recommendation [18]. The timing of introduction of complementary feeding may be influenced by several factors, including sociodemographic (e.g., parental education level, age, place of residence, maternal smoking behavior) and birth-related [18,19,20,21,22].

The primary aim of this study was to determine sociodemographic and birth-related factors associated with the age of introducing complementary foods (<4 months; 4–6 months; >6 months). Secondary aims were to describe the differences in the age of complementary feeding introduction between Poland and Austria.

## 2. Materials and Methods

### 2.1. Study Design

This cross-sectional study investigated Polish and Austrian parents of children aged 12–36 months using a single online questionnaire in January 2017–January 2019. This study was conducted according to the principles of the Declaration of Helsinki. In accordance with Polish and Austrian law, it did not need official ethics approval. The online questionnaire was preceded by information about the study, the voluntariness of participation, and the possibility of resigning from the participation at any stage of questionnaire completing without saving any responses. All data obtained from the online form were collected anonymously, and we did not collect the IP address.

### 2.2. Study Group

**Figure 1:**
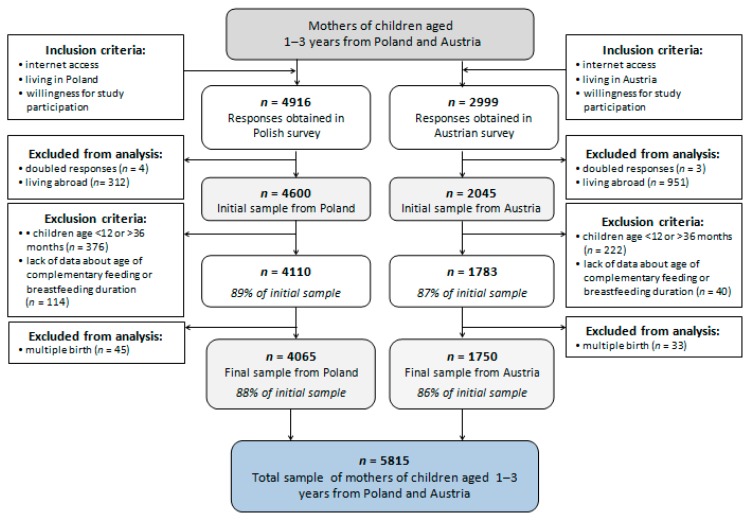
Study sample collection flowchart.

Participants were recruited mainly through Facebook and parenthood-specific discussion boards. In advertising materials, this study was named “feeding of children aged 1–3 years”, and the terms baby-led weaning, breastfeeding, and complementary feeding were avoided to prevent any recruitment bias. Both language versions of the questionnaire (Polish and German) were administered using the online tool Google Forms. The inclusion criteria were internet access, willingness for study participation, living in Poland or Austria, and being the parent or legal guardian of children aged 12–36 months, whereas exclusion criteria were not responding to the questions about complementary feeding or breastfeeding. Parents with more than one child meeting these criteria were asked to complete the survey on one occasion only for one child. The 36-month cut-off point was applied to facilitate recruitment whilst decreasing inaccurate answers due to fading memories. Both language versions of the questionnaire were completed by 7915 participants, and finally, the analysis was conducted among 5815 participants (Figure 1). The total number of participants was higher in Poland than in Austria, due to differences in the population size of both countries (38.0 million in Poland vs. 8.8 million in Austria) [23].

### 2.3. Questionnaire

#### 2.3.1. Demographics Data

The following sociodemographic data were collected on each participating family: maternal and paternal age and educational level, living area (number of inhabitants and voivodeship in Poland and postal code in Austria which was transformed into rural and urban place of living), average monthly income per capita in the household, and number of adults and children living in the household.

Based on the obtained data, selected variables were categorized. Place of residence was categorized according to the living area: (1) rural or (2) urban (towns and cities). Moreover, place of residence was categorized by gross domestic product (GDP) per inhabitant, expressed as a percentage of the EU-28 index based on GDP per capita in purchasing power standards in relation to EU-28 average [24]: (1) 47%–50% (Polish voivodships: Holy Cross, Podlaskie, Lublin, Subcarpathian, Warmian-Masurian); (2) 51%–100% (Polish voivodships: Lower Silesian, Kuyavian-Pomeranian, Lubusz, Łódź, Lesser Poland, Opole, Pomeranian, Silesian, Greater Poland, West Pomeranian; Austrian region: Burgenland); (3) 101%–110% (Polish voivodship: Masovian; Austrian regions: Kärnten, Niederösterreich); (4) 111%–130% (Austrian regions: Oberösterreich, Steiermark); (5) 131%–150% (Austrian regions: Tirol, Vorarlberg); (6) >150% (Austrian regions: Wien, Salzburg). Maternal and paternal age were categorized from quantitative variables as follows: (1) < 25 years; (2) 25–29 years; (3) 30–34 years; (4) 35–39 years; and (5) ≥40 years. Maternal and paternal educational levels were transformed into three categories: (1) primary and vocational certificate, (2) high school, and (3) university degree.

The following demographic data were collected for the children: current age in months, sex, gestational age, type of pregnancy (singleton or multiple), and birth parameters, including birthweight. Children born from multiple pregnancies were excluded from analysis due to a low number of cases in the study group (Figure 1). Children were grouped into three categories according to gestational age: (1) preterm birth (<37 weeks of gestation), (2) term birth (≥37–42 weeks of gestation), and (3) post-term birth (>42 weeks of gestation). Birthweight to gestational age centiles was calculated based on the data about birthweight, gestational age, and INTERGROWTH-21st standards [25] using the INTERGROWTH-21st Neonatal Size Calculator [26]. Small to gestational age (SGA) was defined as birthweight to gestational age lower than the 10th percentile, appropriate to gestational age as 10th–90th percentile, and large to gestational age as >90th percentile.

#### 2.3.2. Milk Feeding Practices

Mothers were also asked whether their children were ever breastfed and about exclusive as well as partial breastfeeding duration in months. Exclusive breastfeeding was defined according to the WHO definition, which assumes that children are not allowed to receive other food or drinks, including water, except breastmilk, but it is allowed to receive vitamins, minerals, and medicines [4]. Mothers were also asked about formula feeding, including receiving formula at the maternity ward. Due to the WHO definition of exclusive breastfeeding, children who received infant formula at the maternity ward were categorized as non-exclusively breastfed.

#### 2.3.3. Introduction of Solids and First Food Offered, Family Food Environment, and Meal Patterns

Parents were also asked about children´s feeding practices in the first 3 months of complementary feeding when the first liquid or solids (besides breastmilk or infant formula) were introduced, developmental signs of readiness for solids, method of complementary feeding (traditional spoonfeeding (TSF), baby-led weaning (BLW), and partially BLW). The age of complementary feeding was reported in chronological age (months) and among prematurely born infants was expressed in corrected age. The quantitative variable was categorized as follows: (1) complementary feeding started before 4 months, (2) complementary feeding started between 4 and 6 months, and (3) complementary feeding started after completed 6 months after birth. TSF was defined as only or mostly spoonfeeding by an adult. Partially BLW was defined as spoonfeeding by an adult half of the time, and baby feeding themselves the other half of the time. BLW was defined as baby feeding themselves all the time. Parents were also asked about the type of food that infants consumed most often during the first three months of complementary feeding (ready-to-eat, homemade especially for infants, family food), as well as the first products and meals consumed by the infant. Frequency of consumed meals and environmental conditions during meal consumption, as well as sources of knowledge about child nutrition were also examined.

#### 2.3.4. Toddlers’ Dietary Habits

We also asked about the last 3 months in toddlers’ feeding behavior: frequency of selected foodstuffs consumed, according to following categories: (1) never or almost never; (2) less than 1 time per week; (3) 1 time per week; (4) at least 2–4 times per week; and (5) 1 time per day. Mothers were also asked about using salt and sugar, methods of consuming meals, and willingness for the consumption of different food consistencies.

#### 2.3.5. Toddlers’ Health and Development

Parents were also asked about toddlers’ current body weight and height; based on these data, the BMI z-score was calculated and interpreted based on WHO criteria [27]. We also asked about the occurrence of sensory integration disorders or food allergies, and developmental skills, including gross and fine motor and language development.

### 2.4. Statistical Analysis

The analysis was conducted using STATISTICA 13.3 (Dell Inc., Tulusa, OK, USA) software. For nominal variables, a chi-square test was performed, and results were expressed as percentage and number. The factors associated with the age of introduction of complementary feeding were investigated using univariate and multivariate logistic regression analysis, which was conducted for the total sample, as well as for the Polish and Austrian samples separately. Each category of age during starting complementary feeding was coded as binary for the purpose of these analyses. The models included sociodemographic variables (maternal age and educational level, number of children in the family, average monthly income per capita, macroeconomic region, living area) and variables related to pregnancy and early postnatal development (pregnancy duration, birthweight to gestational age, any breastfeeding and receiving of infant formula). Moreover, an additional analysis of factors influencing too early introduction of complementary foods was conducted separately in preterm and term infants. For the purposes of logistic regression analysis, variables such as pregnancy duration and receiving infant formula at maternity ward were recategorized (preterm vs. term birth; no infant formula vs. otherwise, respectively) due to a low number of cases in those categories. For all analyses, *p* ≤ 0.05 was considered significant.

## 3. Results

### 3.1. Study Group Characteristics

#### 3.1.1. Family Characteristics

Maternal and family characteristics are shown in Table 1. Around 45% of parents from both countries were aged 30–34 years, whereas Austria’s parents tended to be older (*p* ≤ 0.001). Most parents also had a university education, a higher percentage of mothers compared to fathers (82.2% vs. 61.7%), and a higher rate of university education was observed in Poland compared to Austria. Households with 3 people and 1 child among them dominated in study groups, especially in Poland (62.3% vs. 47.1% and 67.8% vs. 48.9%, respectively). More than two-thirds of the respondents lived in an urban area, more often in Poland than in Austria, but respondents in Austria lived in regions with a higher GDP EU-28 average than those in Poland, as well as had a higher average monthly income per capita.

We also observed diversity in sociodemographic factors according to the age of introducing complementary feeding (Table S1). Parents who introduced complementary feeding earlier were younger and had a lower education level. Higher socioeconomic disparities were observed in Poland compared to Austria, where differences in variables such as maternal age, parental age and education, household size, living area, and average monthly income were not statistically significant.

#### 3.1.2. Children’s Characteristics

The average age of the children was 22.4 months, and just over half of the children were male (51.6%). Most of the children were born at term (93.6%) and had an appropriate birthweight to gestational age (77.8%). In Poland, children who participated in the study were slightly younger (21.8 vs. 23.9 months, *p* ≤ 0.001), and higher rates of LGA were observed (19.1% vs. 13.2%, *p* ≤ 0.001; Table S2). Most of the children were breastfed (93.7%), but also half of the children received infant formula at the maternity ward (50.3%), and one-third received infant formula after discharge (32.0%). In Poland, higher rates of any breastfeeding (94.7% vs. 91.4%, *p* ≤ 0.001), as well as receiving infant formula both at the maternity ward (55.6% vs. 38.1%, *p* ≤ 0.05) but the same after discharge (32.0%) were observed.

#### 3.1.3. Age of Introduction of Complementary Feeding

More than half of the children (65.0%) were introduced to complementary foods between 4 and 6 months, whereas introducing after 6 completed months was observed in 32.1% and early (<4 months) in 3.0% of children. More than one-quarter of the children were introduced to complementary foods using the traditional spoonfeeding method (30.7%), around half using the partially BLW method (53.0%), and 16.3% using the BLW method. In Austria, complementary feeding was introduced earlier and more often using the TSF method (Table S2). Formula-fed infants were introduced to complementary foods earlier than breastfed infants: before 4 months 8.8% vs. 2.6%, between 4 and 6 months 76.7% vs. 64.2%, and after 6 completed months 14.5% vs. 33.2% (*p* ≤ 0.001).

In children who were introduced to complementary foods early (<4 months), higher average child age was observed, as well as rates of premature births, SGA, receiving infant formula at the maternity ward and after discharge, as well as lower rates of any breastfeeding. Further, children who were introduced to complementary foods earlier were weaning using the TSF method, whereas infants who were introduced to complementary foods after 6 completed months were introduced to foods using the BLW method. These differences were similar in Poland and Austria despite the children’s age, category of birthweight to gestational age, and any breastfeeding rates, which were not observed in Austria (Table 2).

### 3.2. Factors Associated with Complementary Feeding Start before 4 Months

The results of the logistic univariate and multivariate logistic regression analysis conducted separately in Poland and Austria are shown in Table 3. An analysis conducted in Poland showed that the odds of being introduced to complementary foods early (<4 months) were higher among children of mothers with lower education, as well as in children born prematurely and who were never breastfed or never received infant formula after discharge. Data from Austria showed a higher odds among children of mothers aged 25–29 years old, with lower education and living in an area with GDP >150% EU-28 average, as well as in children born prematurely and who received infant formula after hospital discharge.

In addition, Table S3 presents logistic regression models investigating factors associated with the early introduction of complementary feeding conducted separately among preterm- and term-born infants. Factors increasing the odds of too early complementary feeding among preterm infants were lower maternal education, living in Austria, lack of breastfeeding, and formula feeding after hospital discharge. The odds of introducing complementary foods before 4 months decreased by 33% to every additional week of pregnancy duration. In term infants, those risk factors were lower maternal age, but also age 35–39 years, and education, living in Austria, and formula use after discharge, while formula use at maternity ward decreased those odds.

### 3.3. Factors Associated with Complementary Feeding Start between 4 and 6 Months

The odds of being introduced to complementary foods between 4 and 6 months in Poland were higher among children of mothers younger than 30 years, never breastfed or who received infant formula at the maternity ward and after hospital discharge (Table 4). In Austria, by contrast, these odds were higher among children born in families living in a rural area, a region with 131%–150% GDP EU-28 average, and who received infant formula after hospital discharge. This odd was lower if there were 3 or more children in the household or children who were breastfed or born prematurely.

### 3.4. Factors Associated with Complementary Feeding Start after 6 Completed Months

The odds of being introduced to complementary foods after 6 completed months in Poland were lower among children of mothers younger than 30 years and lower-educated, living in a region with GDP level at 47%–50% of EU-28 average, born preterm or SGA, among never breastfed infants and children who received infant formula after discharge (Table 5).

In Austria, the odds of being introduced to complementary foods after 6 completed months were higher among children with at least two siblings. Lower odds were observed in children living in rural areas, a region with GDP level at 131%–150% of EU-28 average, born prematurely, never breastfed or receiving infant formula after hospital discharge.

## 4. Discussion

This study has shown that complementary foods were introduced early (<4 months) in 3.0% of infants from both countries, but in a higher proportion in the Austrian sample compared to the Polish (2.4% vs. 4.3%), as well as formula-fed infants compared to breastfed (5.3% vs. 8.6%). The national recommendation of complementary feeding between 17 and 26 weeks of infant age was followed by 65.0% of the study group. On the other hand, the WHO recommendation of introducing complementary feeding after 6 completed months was followed by 32.1% of the study group, more often in Poland than Austria (37.1% vs. 20.4%). Our study also showed that the strongest sociodemographic factors related to an earlier introduction than recommended by WHO of complementary feeding were lower maternal age and education, but factors such as living area or parity also influenced this decision. Among pregnancy-related factors, the strongest determinants of timing of complementary feeding were preterm birth and, to a lesser extent, birthweight to gestational age, whereas among feeding-related factors, never breastfeeding and receiving an infant formula after hospital discharge were the strongest determinants.

### 4.1. Age of Introduction of Complementary Feeding

The rates of introducing complementary feeding before 4 completed months of life observed in our study (3.0%) were lower than worldwide and the European estimated average (11% and 5%, respectively) [17]. However, the IDEFICS (Identification and prevention of Dietary- and lifestyle-induced health Effects in Children and infantS) study conducted in eight European countries showed higher rates of early introduction of solids (12.2%) [28]. Previous studies reported the highest percentage of early introduction to solids in Australia (43.5% [29]), the UK (36% [30]), Netherlands (21.4% [31]), and the USA (16.3% [32]). By contrast, these rates were the lowest in Italy and Scandinavia (7% [33] and around 5% [20,22,34], respectively). In Germany (Bavaria), 16% of infants were already introduced to complementary foods at the beginning of the fifth month [35]. Compliance with the WHO recommendations was higher in our study (32.1%) than that observed by other authors in Norway (14.2% [22]), Italy (14% [33]), the USA (12.9% [32]), Denmark (10% [20]), the European study IDEFICS (11.9% [28]), and Sweden (3.7% [34]). However, the proportions of infants who received their first complementary foods after 6 completed months were higher in the Netherlands (38% [19]).

### 4.2. Sociodemographic Factors Related to the Age of Introduction of Complementary Feeding

The most important sociodemographic factors that influenced early complementary feeding were lower education and, to a lesser extent, maternal age. Our results confirmed that younger mothers tend to introduce solids earlier (in Austria before 4 months, in Poland between 4 and 6 months), which was shown in previous research conducted in Europe [18,19,22,31,35], the USA [32], and Australia [29]. In our study, lower maternal education was a factor influenced only by early (<4 months) introduction of complementary feeding, which is in line with previous studies [18,22,31,35]. However, some studies found that education level was inversely associated with the age of complementary feeding only with mothers of Western or Caucasian origin [19,30]. In our study, multiple parities were a factor that decreased the odds of complementary feeding between 4 and 6 months and increased the odds of starting it after 6 completed months in Austria. These observations are in contrast with the results of the study conducted in the Netherlands, where multiple parities increased the odds of complementary feeding between 3 and 6 months [19]. Economic difficulties are also a factor that may predispose to introducing solids earlier than recommended [18,22]. In contrast to previous studies in our study, the average monthly income did not influence the age of introducing complementary feeding, which was also observed before in the Netherlands [19]. However, living in a region with a lower GDP than the EU-28 average decreased the odds of complementary feeding after 6 completed months in Poland. On the other hand, living in the area with a higher GDP than the EU-28 average increased the odds of complementary feeding starting before 4 months in Austria, as well as was related to decreased odds of complementary feeding after 6 completed months in Austria. Other maternal factors not investigated in this study also may influence infant feeding practices. Previous research has shown that early maternal return to work [30], tobacco smoking [22,29,35], and symptoms of anxiety and depression [22] led to an earlier introduction of complementary feeding.

### 4.3. Pregnancy-Related Factors Related to the Age of Introduction of Complementary Feeding

Regarding pregnancy-related factors, the study revealed that the strongest factor related to the earlier than 6 completed months introduction of complementary feeding observed in our study was preterm birth. This is consistent with the observations of other authors [36,37]. Moreover, Braid et al. [37] showed that more premature infants born at 22 to 32 weeks’ gestation were at greater risk of early solids introduction compared to later-born preterm (33 to 36 weeks) and term infants. We also found that every week of pregnancy duration was associated with a decrease of 33% of odds of early introduction to solids. On the other hand, a study of late preterm infants from Italy showed that in most cases, infants were introduced to complementary foods within 4 and 6 months of postnatal age [38]. Currently, there is no specific recommendation about introducing complementary feeding in prematurely born infants; nonetheless, it should be considered due to increased nutritional requirements, and the possibility of delayed motoric development, feeding difficulties or respiratory compromise depending on gestational age or comorbid diseases [37]. A study performed among Italian primary care pediatricians showed that most of them believe that the decision about introducing solids into an infant’s diet should be not only based on infant age (44%) but also based on neurodevelopmental status (18%), infant body mass (4%), or at least two factors collectively [39]. Earlier introduction to solids in preterm born infants compared to term-born may be caused by using parents’ chronological age, not the corrected one, when making this decision [37]. Preterm-born infants have a higher risk of iron deficiency [40]. The ESPGHAN recommends giving infants from the risk groups iron supplements and, from the age of 6 months, iron-rich foods [41]. However, EFSA states that some exclusively breastfed infants aged 4 to 6 months may require complementary foods earlier due to iron deficiency [10]. It was reported previously that parents who introduced complementary foods earlier heard from “a doctor or other health professional that their infant should begin eating solid food” or their infants ”had a medical condition that might be helped by eating solids” [38,42]. Nonetheless, it should be emphasized that the earlier introduction of complementary feeding into a diet of prematurely born infants resulted in a higher rate of hospitalizations [16].

In our study, we did not observe any association between birthweight to gestational age and the timing of introducing solids. Contradictory to our results, Fewtrell et al. [36] showed that small to gestational age term infants had a lower risk of early introduction to solids compared to infants born with an adequate birthweight. The authors explained that this may reflect parental concern on infant vulnerability and higher adherence to nutritional recommendations.

### 4.4. Children-Feeding Factors Related to the Age of Introduction of Complementary Feeding

The current study showed that any breastfeeding and introduction of formula into infant diet after hospital discharge were important factors increasing the risk of early introduction of complementary foods. Our results are in line with previously conducted studies, which found that infants introduced to solids earlier were less often exclusively breastfed or more often were partly breastfed for a shorter time or never [18,19,20,22,29,31,32,35,36,37,42]. The possible explanation may be that mothers who never initiated breastfeeding or breastfed for a shorter time (exclusive or any breastfeeding) have a lower educational level, nutritional knowledge, lack of knowledge about proper infant nutrition and/or less interest in infant nutrition [35]. On the other hand, a study conducted by Clayton et al. [42] showed that parents of formula-fed infants more often heard from relatives or family and health professionals that infants should begin eating solids compared to mixed or exclusively breastfed infants. Mothers who formula-fed their infants and introduced solids before 4 months also more often thought that infants were “drinking too much formula” or that their “baby seemed hungry all the time” [42].

Another important factor that may compromise the timing of complementary feeding is the marketing of commercially available complementary foods. A lot of them are labeled as good for infants starting from 4 months, which may encourage the earlier introduction of complementary foods, contrary to WHO recommendations [43]. Marketing strategies used by manufacturers can confuse and mislead caregivers about the nutritional qualities of these types of foods, as well as about the age of introduction, and may intensify maternal concerns about the adequacy of breastfeeding [43,44]. On the other hand, early complementary foods have a lower nutrient- and energy-density than breastmilk or infant formula, which may lead to nutritional deficiencies [9,43]. Early introduction of complementary foods may result in a decrease of the amount of consumed milk [36] and in consequence results in a shorter duration of breastfeeding [9].

Guidelines for complementary feeding have changed over the last few years. Previously, there were different recommendations about the timing of complementary feeding for formula- and exclusively breastfed infants, as well as specific timing of gluten introduction. Currently, Polish and Austrian recommendations are that infants should be exclusively breastfed for around 6 months, while complementary feeding recommendations state that complementary foods should be introduced between 17 and 26 weeks of life. This may lead to confusion for caregivers, or even some health professionals, about the appropriate timing of starting to introduce complementary foods. The necessity of better education of health professionals for giving caregivers evidence-based advice and support of exclusively breastfeeding for 6 months is emphasized. Moreover, given the increasing amount of evidence that early nutrition has marked effects both on short- and long-term children’s health, further educational programs, and guidance should focus on improving complementary feeding practices especially in the risk groups.

### 4.5. Strengths and Limitations

The main strength of this study was, first, the large sample size (*n* = 5815) from two European countries not limited to one place of residence. Second, there is a small number of large nationwide studies that have investigated this area in Poland and Austria [18]. Third, thanks to the study design and web administration of the questionnaire, it may be possible to decrease the social desirability bias in the obtained answers. Fourth, this study was able to identify several potential factors from three different areas (sociodemographic characteristics, pregnancy outcomes, and early feeding practices) associated with the timing of the introduction of complementary feeding. It is worth noticing that some of these factors, such as breastfeeding initiation and formula feeding, may be modifiable.

The current study also had some limitations. First, the infant age during the introduction of complementary feeding was self-reported by parents retrospectively when children were between 12 and 36 months of life. Second, the participants were only limited to internet users. However, in Poland, the number of internet users has been systematically increasing in recent years, reaching up to 83% in 2018, especially in households with children, where 99% of households have internet access. In Austria, internet access is available in 88% of households [45,46]. Third, the used method of questionnaire distribution did not allow for recording unfinished questionnaires, so the number of participants who started the study without finishing it is unknown, as well as their sociodemographic characteristics. Fourth, there is the possibility that parents interested in children’s nutrition and in consequence following the nutritional recommendations finished the study more often. This may result in underestimation of the percentage of infants who received complementary feeding before 4 months, which should be taken into account in the interpretation of the results. Despite the mentioned limitations, this study provides interesting results that are relevant for designing educational programs and interventions.

## 5. Conclusions

This study identified important maternal and pregnancy-related determinants for the timing of the introduction of complementary feeding. Our study showed that factors associated with inappropriate timing of introducing complementary feeding were younger maternal age and education level, preterm birth, lack of breastfeeding initiation, and formula feeding. Moreover, we found that in preterm infants, the risk of a too-early introduction of solids decreased with the duration of pregnancy. The results of this study are in line with the results of previous studies conducted in developed countries and may be used for the establishment of educational programs and nutritional guidelines. These factors should be taken into account by health professionals in identifying mothers who are least likely to follow nutritional recommendations.

## Figures and Tables

**Table 1:** Study group sociodemographic characteristics.

Variable	Poland (*n* = 4065) % (n)	Austria (*n* = 1750) % (n)	*p*-Value
**Maternal age:**			≤0.001
<25 years	4.7 (191)	4.6 (80)	
25–29 years	32.5 (1320)	26.1 (457)	
30–34 years	47.7 (1938)	39.5 (692)	
35–39 years	13.6 (554)	24.9 (436)	
≥40 years	1.5 (62)	4.9 (85)	
**Paternal age:**			≤0.001
<25 years	1.5 (59)	2.0 (35)	
25–29 years	20.2 (823)	14.3 (250)	
30–34 years	48.8 (1985)	34.2 (598)	
35–39 years	22.7 (924)	30.3 (531)	
≥40 years	6.7 (274)	19.2 (336)	
**Maternal education:**			≤0.001
primary and vocational	0.9 (38)	26.2 (458)	
high school	11.7 (477)	3.6 (63)	
University	87.3 (3550)	70.2 (1229)	
**Paternal education:**			≤0.001
primary and vocational	9.1 (373)	45.0 (787)	
high school	23.4 (950)	6.6 (116)	
University	67.5 (2742)	48.4 (847)	
**Household size:**			≤0.001
2	2.0 (81)	2.2 (39)	
3	62.3 (2531)	47.1 (824)	
4	28.3 (1149)	37.8 (661)	
5	5.5 (225)	9.0 (158)	
≥6	1.9 (79)	3.9 (68)	
**Number of children living in the household:**			≤0.001
1	67.8 (2758)	48.9 (855)	
2	27.8 (1129)	36.6 (641)	
≥3	4.4 (178)	14.5 (254)	
**Living area:**			≤0.001
rural	17.3 (705)	56.3 (986)	
urban	82.7 (3360)	43.7 (764)	
**Living macroeconomic region:**			≤0.001
47%–50% GDP EU-28 average	13.9 (564)	-	
51%–100% GDP EU-28 average	62.7 (2550)	5.5 (96)	
101%–110% GDP EU-28 average	23.4 (951)	42.1 (737)	
111%–130% GDP EU-28 average	-	26.2 (459)	
131%–150% GDP EU-28 average	-	10.2 (179)	
>150% GDP EU-28 average	-	15.9 (279)	
**Average monthly income per capita ^1^:**			≤0.001
1st category	1.5 (59)	8.5 (149)	
2nd category	14.5 (588)	20.8 (364)	
3rd category	32.7 (1328)	31.4 (550)	
4th category	17.4 (706)	26.4 (462)	
5th category	12.3 (499)	10.6 (186)	
6th category	21.8 (885)	2.2 (39)	

^1^ Average monthly income per capita categories depends on country: 1st category <500 PLN (Poland) or <1000 EUR (Austria); 2nd category 500–1000 PLN/1000–1500 EUR; 3rd category 1001–2000 PLN/1501–2000 EUR; 4th category 2001–2500 PLN/2001–3000 EUR; 5th category 2501–3000 PLN/3001–5000 EUR; 6th category ≥3001 PLN/≥5001 EUR.

**Table 2:** Children’s characteristics including birth parameters and feeding methods according to the age of introducing complementary feeding into infant diet.

Variable	Poland (*n* = 4065) Mean ± SD ^1^ Min–Max % (*n*)				Austria (*n* = 1750) Mean ± SD Min–Max % (*n*)			
	<4 mo	4–6 mo	>6 mo	*p*-Value	<4 mo	4–6 mo	>6 mo	*p*-Value
**Infant age:**	24.2 ± 7.6	22.1 ± 7.3	21.3 ± 7.1	≤0.01	25.1 ± 8.1	23.9 ± 7.7	23.7 ± 7.9	0.110
	12.0–36.0	12.0–36.0	12.0–36.0		12.0–36.0	12.0–36.0	12.0–36.0	
**Infant gender:**				0.085				0.468
female	52.0 (51)	46.3 (1139)	49.6 (748)		44.7 (34)	49.8 (656)	52.4 (187)	
male	48.0 (47)	53.7 (1321)	50.4 (759)		55.3 (42)	50.2 (661)	47.6 (170)	
**Pregnancy duration:**								
<37 weeks	79.6 (78)	7.2 (178)	0.3 (4)	≤0.001	59.2 (45)	4.4 (58)	2.0 (7)	≤0.001
37–42 weeks	19.4 (19)	92.2 (2268)	99.3 (1497)		39.5 (30)	94.6 (1246)	98.0 (350)	
>42 weeks	1.0 (1)	0.6 (14)	0.4 (6)		1.3 (1)	1.0 (13)	-	
**Birthweight to gestational age categories:**								
SGA ^2^	7.1 (7)	4.6 (113)	3.1 (47)	0.016	11.8 (9)	6.2 (81)	7.8 (28)	0.293
AGA ^3^	68.4 (67)	75.9 (1866)	79.0 (1190)		73.7 (56)	80.8 (1064)	78.7 (281)	
LGA ^4^	24.5 (24)	19.5 (481)	17.9 (270)		14.5 (11)	13.0 (172)	13.5 (48)	
**Any breastfeeding:**								
no	22.5 (22)	7.2 (178)	1.0 (15)	≤0.001	13.2 (10)	7.7 (102)	10.6 (38)	0.076
yes	77.5 (76)	92.8 (2282)	99.0 (1492)		86.8 (686)	92.3 (1215)	89.4 (319)	
**Infant formula use at maternity ward:**								
no	28.6 (28)	36.9 (908)	47.2 (711)	≤0.001	36.8 (28)	61.0 (804)	67.8 (242)	≤0.001
don’t know	3.1 (3)	3.8 (94)	4.1 (62)		1.3 (1)	0.5 (6)	0.6 (2)	
yes	68.4 (67)	59.3 (1458)	48.7 (734)		61.8 (47)	38.5 (507)	31.7 (113)	
**Infant formula use after discharge:**								
no	34.7 (34)	61.5 (1512)	80.9 (1219)	≤0.001	38.2 (29)	67.0 (882)	78.2 (279)	≤0.001
yes	65.3 (64)	38.5 (948)	19.1 (288)		61.8 (47)	33.0 (435)	21.8 (78)	
**Complementary feeding method:**								
TSF ^5^	60.2 (59)	35.9 (884)	13.3 (201)	≤0.001	52.6 (40)	39.5 (520)	22.1 (79)	≤0.001
mixed	35.7 (35)	52.8 (1299)	58.7 (884)		39.5 (30)	49.2 (648)	52.4 (187)	
BLW ^6^	4.1 (4)	11.3 (277)	28.0 (422)		7.9 (6)	11.3 (149)	25.5 (91)	

^1^ SD—standard deviation; ^2^ SGA—small to gestational age; ^3^ AGA—appropriate to gestational age; ^4^ LGA—large to gestational age; ^5^ TSF—traditional spoonfeeding; ^6^ BLW—baby-led weaning.

**Table 3:** Univariate and multivariate regression analysis models of factors influencing the complementary feeding start before 4 months.

	Variable	Poland (*n* = 4065)		Austria (*n* = 1750)	
		OR ^1^ (95% CI ^2^)	aOR ^3^ (95% CI)	OR (95% CI)	aOR (95% CI)
SOCIODEMOGRAPHIC FACTORS	**Maternal age:**				
	<25 years	2.41 (1.15–5.05) *	1.17 (0.37–3.73)	2.24 (0.82–6.14)	2.49 (0.76–8.11)
	25–29 years	1.29 (0.81–2.05)	1.64 (0.90–2.98)	1.94 (1.07–3.54) *	2.21 (1.06–4.65) *
	30–34 years	1	1	1	1
	35–39 years	1.26 (0.68–2.34)	1.33 (0.62–2.89)	1.87 (1.02–3.45) *	1.76 (0.83–3.71)
	≥40 years	1.62 (0.38–6.88)	1.37 (0.22–8.32)	1.23 (0.36–4.23)	0.65 (0.14–2.92)
	**Maternal education:**				
	primary and vocational	8.93 (3.62–22.02) ***	14.49 (3.73–56.35) ***	1.62 (0.99–2.67)	2.13 (1.10–4.11) *
	high school	1.98 (1.18–3.30) **	2.20 (1.06–4.60) *	2.84 (1.16–6.93) *	3.35 (1.09–10.28) *
	university	1	1	1	1
	**Number of children in household:**				
	1	1.16 (0.73–1.86)	0.93 (0.52–1.69)	1.45 (0.86–2.46)	0.79 (0.42–1.51)
	2	1	1	1	1
	≥3	1.61 (0.65–3.99)	1.53 (0.43–5.50)	1.40 (0.68–2.86)	1.07 (0.46–2.49)
	**Living area:**				
	rural	1.00 (0.59–1.70)	0.59 (0.30–1.15)	1.31 (0.71–1.81)	1.41 (0.68–2.92)
	urban	1	1	1	1
	**Living in macroeconomic region** (GDP EU-28 average):				
	47–50%	1.79 (1.01–3.17) *	1.69 (0.77–3.67)	-	-
	51–100%	0.76 (0.46–1.24)	0.84 (0.45–1.56)	1.34 (0.51–3.55)	1.36 (0.43–4.23)
	101–110%	1	1	1	1
	111–130%	-	-	1.05 (0.58–1.90)	0.89 (0.44–1.81)
	131–150%	-	-	0.85 (0.35–2.07)	0.94 (0.32–2.79)
	>150%	-	-	1.58 (0.86–2.93)	2.52 (1.02–6.26) *
	**Average monthly income per capita ^4^:**				
	1st category	1.42 (0.32–6.31)	0.70 (0.10–4.80)	1.37 (0.55–3.41)	1.01 (0.34–3.03)
	2nd category	1.58 (0.83–3.00)	1.08 (0.44–2.64)	1.45 (0.73–2.89)	1.09 (0.47–2.51)
	3rd category	0.71 (0.38–1.35)	0.55 (0.25–1.19)	1.11 (0.57–2.15)	1.02 (0.46–2.28)
	4th category	1	1	1	1
	5th categery	0.58 (0.24–1.40)	0.16–1.24)	1.92 (0.89–4.15)	2.28 (0.91–5.72)
	6th category	1.23 (0.69–2.36)	1150.53–2.50)	1.51 (0.33–6.81)	1.01 (0.14–7.08)
PREGNANCY-RELATED FACTORS	**Pregnancy duration:**				
	preterm	8.11 (4.86–13.55) ***	10.21 (5.73–18.20) ***	3.59 (2.14–6.05) ***	4.45 (2.42–8.18) ***
	term	1	1	1	1
	**Birthweight to gestational age categories:**				
	SGA ^5^	2.00 (0.90–4.42)	1.33 (0.46–3.84)	1.98 (0.96–4.12)	1.30 (0.55–3.10)
	AGA ^6^	1	1	1	1
	LGA ^7^	1.46 (0.91–2.34)	1.74 (0.97–3.13)	1.20 (0.62–2.33)	1.59 (0.71–3.53)
FEEDING-RELATED FACTORS	**Any breastfeeding:**				
	no	5.66 (3.45–9.30) ***	2.73 (1.29–5.76) **	1.66 (0.84–3.30)	1.49 (1.20–2.21)
	yes	1	1	1	1
	**Infant formula use at maternity ward:**				
	no	1	1	1	1
	otherwise	1.72 (1.11–2.68) *	0.61 (0.33 – 1.21)	2.86 (1.77–4.60) ***	1.24 (0.64–2.41)
	**Infant formula use after discharge:**				
	no	1	1	1	1
	yes	4.16 (2.73–6.34) ***	3.73 (2.06 – 6.75) ***	3.67(2.28–5.89) ***	3.65 (1.87–7.12) ***

^1^ OR—univariate model; ^2^ CI—confidence intervals; ^3^ aOR—multivariate model; ^4^ Average monthly income per capita categories depends on country: 1st category <500 PLN (Poland)/<1000 EUR (Austria); 2nd category 500–1000 PLN/1000–1500 EUR; 3rd category 1001–2000 PLN/1501–2000 EUR; 4th category 2001–2500 PLN/2001–3000 EUR; 5th category 2501–3000 PLN/3001–5000 EUR; 6th category ≥3001 PLN/≥5001 EUR; ^5^ SGA—small to gestational age; ^6^ AGA—appropriate to gestational age; ^7^ LGA—large to gestational age; * *p*-value ≤ 0.05; ** *p*-value ≤ 0.01; *** *p*-value ≤ 0.001.

**Table 4:** Univariate and multivariate regression analysis models of factors influencing the complementary feeding introduction at the age of 4–6 months.

	Variable	Poland (*n* = 4065)		Austria (*n* = 1750)	
		OR ^1^ (95% CI ^2^)	aOR ^3^ (95% CI)	OR (95% CI)	aOR (95% CI)
SOCIODEMOGRAPHIC FACTORS	**Maternal age:**				
	<25 years	1.75 (1.27–2.42) ***	1.57 (1.10–2.25) *	0.65 (0.39–1.08)	0.60 (0.35–1.02)
	25–29 years	1.39 (1.20–1.61) ***	1.34 (1.15–1.57) ***	0.91 (0.69–1.20)	0.89 (0.67–1.19)
	30–34 years	1	1	1	1
	35–39 years	1.03 (0.85–1.25)	1.01 (0.83–1.23)	0.84 (0.63–1.10)	0.85 (0.63–1.13)
	≥40 years	0.85 (0.51–1.41)	0.82 (0.49–1.38)	0.80 (0.48–1.33)	0.88 (0.52–1.51)
	**Maternal education:**				
	primary and vocational	1.30 (0.66–2.55)	0.98 (0.49–1.98)	0.99 (0.77–1.27)	0.93 (0.71–1.23)
	high school	1.33 (1.08–1.62) **	1.19 (0.96–1.49)	0.60 (0.35–1.02)	0.60 (0.34–1.05)
	University	1	1	1	1
	**Number of children in household:**				
	1	1.02 (0.89–1.18)	0.90 (0.77–1.05)	0.97 (0.76–1.24)	1.02 (0.79–1.32)
	2	1	1	1	1
	≥3	0.75 (0.54–1.03)	0.84 (0.61–1.17)	0.66 (0.48–0.91) *	0.69 (0.49–0.96) *
	**Living area:**				
	rural	1.22 (1.03–1.45) *	1.13 (0.95–1.35)	1.23 (0.99–1.53)	1.38 (1.06–1.80) *
	urban	1	1	1	1
	**Living in macroeconomic region (GDP EU-28 average):**				
	47–50%	1.32 (1.06–1.64) *	1.20 (0.96–1.52)	-	-
	51–100%	0.97 (0.83–1.13)	0.94 (0.80–1.10)	0.91 (0.56–1.46)	0.90 (0.55–1.47)
	101–110%	1	1	1	1
	111–130%	-	-	1.20 (0.91–1.57)	1.26 (0.95–1.67)
	131–150%	-	-	1.91 (1.24–2.96) **	2.17 (1.38–3.43) ***
	>150%	-	-	0.90 (0.66–1.22)	1.13 (0.79–1.61)
	**Average monthly income per capita ^4^:**				
	1st category	1.34 (0.76–2.34)	0.93 (0.52–1.67)	0.88 (0.58–1.34)	1.00 (0.65–1.56)
	2nd category	1.06 (0.85–1.32)	0.85 (0.67–1.08)	1.02 (0.74–1.40)	1.08 (0.78–1.51)
	3rd category	1.08 (0.90–1.30)	1.01 (0.83–1.22)	1.08 (0.81–1.44)	1.13 (0.84–1.53)
	4th category	1	1	1	1
	5th category	1.09 (0.86–1.38)	1.08 (0.85–1.34)	0.79 (0.54–1.16)	0.89 (0.60–1.32)
	6th category	1.01 (0.82–1.23)	1.03 (0.84–1.27)	0.94 (0.44–1.99)	0.97 (0.45–2.10)
PREGNANCY-RELATED FACTORS	**Pregnancy duration:**				
	preterm	1.45 (1.11–1.90) **	1.27 (0.96–1.68)	0.34 (0.23–0.50) ***	0.31 (0.21–0.47) ***
	term	1	1	1	1
	**Birthweight to gestational age categories:**				
	SGA ^5^	1.41 (1.01–1.97) *	1.37 (0.97–1.93)	0.69 (0.46–1.04)	0.82 (0.54–1.25)
	AGA ^6^	1	1	1	1
	LGA ^7^	1.10 (0.94–1.30)	1.10 (0.93–1.30)	0.92 (0.67–1.27)	0.96 (0.69–1.34)
FEEDING-RELATED FACTORS	**Any breastfeeding:**				
	no	3.31 (2.31–4.74) ***	1.78 (1.21–2.60) **	0.69 (0.48–1.00) *	1.49 (1.31–1.77) **
	yes	1	1	1	1
	**Infant formula use at maternity ward:**				
	no	1	1	1	1
	otherwise	1.46 (1.28–1.66) ***	1.16 (1.01–1.33) *	1.07 (0.85–1.33)	1.07 (0.80–1.43)
	**Infant formula use after discharge:**				
	no	1	1	1	1
	yes	2.23 (1.93–2.58) ***	1.91 (1.63–2.24) ***	1.23 (0.97–1.56)	1.61 (1.17–2.21) **

^1^ OR—univariate model; ^2^ CI—confidence intervals; ^3^ aOR—multivariate model; ^4^ Average monthly income per capita categories depends on country: 1st category <500 PLN (Poland)/<1000 EUR (Austria); 2nd category 500–1000 PLN/1000–1500 EUR; 3rd category 1001–2000 PLN/1501–2000 EUR; 4th category 2001–2500 PLN/2001–3000 EUR; 5th category 2501–3000 PLN/3001–5000 EUR; 6th category ≥3001 PLN/≥5001 EUR; ^5^ SG—small to gestational age; ^6^ AGA—appropriate to gestational age; ^7^ LGA—large to gestational age; * *p*-value ≤ 0.05; ** *p*-value ≤ 0.01; *** *p*-value ≤ 0.001.

**Table 5:** Univariate and multivariate regression analysis models of factors influencing the complementary feeding introduction after 6 completed months.

	Variable	Poland (*n* = 4065)		Austria (*n* = 1750)	
		OR ^1^ (95% CI ^2^)	aOR ^3^ (95% CI)	OR (95% CI)	aOR (95% CI)
SOCIO-DEMOGRAPHIC FACTORS	**Maternal age:**				
	<25 years	0.49 (0.35–0.69) ***	0.58 (0.40–0.85) **	1.34 (0.78–2.30)	1.63 (0.92–2.88)
	25–29 years	0.70 (0.60–0.81) ***	0.72 (0.61–0.84) ***	0.94 (0.70–1.27)	1.01 (0.74–1.38)
	30–34 years	1	1	1	1
	35–39 years	0.95 (0.78–1.15)	0.99 (0.80–1.21)	1.06 (0.79–1.43)	1.11 (0.81–1.52)
	≥40 years	1.13 (0.68–1.88)	1.31 (0.75–2.27)	1.32 (0.78–2.23)	1.39 (0.80–2.41)
	**Maternal education:**				
	primary and vocational	0.36 (0.16–0.83) *	0.51 (0.21–1.21)	0.90 (0.69–1.18)	0.92 (0.68–1.24)
	high school	0.68 (0.56–0.84) ***	0.77 (0.61–0.97) *	1.31 (0.73–2.34)	1.42 (0.76–2.63)
	University	1	1	1	1
	**Number of children in household:**				
	1	0.97 (0.84–1.11)	1.14 (0.97–1.33)	0.95 (0.73–1.23)	1.01 (0.77–1.33)
	2	1	1	1	1
	≥3	1.28 (0.93–1.76)	1.11 (0.79–1.56)	1.48 (1.05–2.08) *	1.55 (1.09–2.21) *
	**Living area:**				
	rural	0.81 (0.69–0.97) *	0.94 (0.78–1.13)	0.77 (0.61–0.98) *	0.66 (0.50–0.88) **
	urban	1	1	1	1
	**Living in macroeconomic region (GDP EU-28 average):**				
	47–50%	0.69 (0.55–0.86) **	0.78 (0.61–0.99) *	-	-
	51–100%	1.06 (0.91–1.24)	1.10 (0.93–1.30)	1.10 (0.67–1.82)	1.19 (0.71–2.00)
	101–110%	1	1	1	1
	111–130%	-	-	0.81 (0.60–1.08)	0.80 (0.59–1.08)
	131–150%	-	-	0.49 (0.30–0.79) *	0.42 (0.25–0.69) ***
	>150%	-	-	1.00 (0.72–1.39)	0.74 (0.51–1.09)
	**Average monthly income per capita ^4^:**				
	1st category	0.71 (0.40–1.26)	1.12 (0.61–2.08)	1.07 (0.69–1.67)	0.98 (0.61–1.56)
	2nd category	0.89 (0.71–1.12)	1.16 (0.90–1.49)	0.90 (0.64–1.26)	0.89 (0.62–1.27)
	3rd category	0.95 (0.79–1.15)	1.04 (0.85–1.27)	0.91 (0.67–1.24)	0.90 (0.66–1.24)
	4th category	1	1	1	1
	5th category	0.96 (0.76–1.22)	0.98 (0.76–1.25)	1.10 (0.73–1.65)	1.02 (0.66–1.56)
	6th category	0.97 (0.79–1.18)	0.96 (0.78–1.20)	0.97 (0.43–2.18)	1.04 (0.45–2.41)
PREGNANCY-RELATED FACTORS	**Pregnancy duration:**				
	preterm	0.02 (0.01–0.06) ***	0.03 (0.01–0.07) ***	0.25 (0.12–0.54) ***	0.26 (0.12–0.57) ***
	term	1	1	1	1
	**Birthweight to gestational age categories:**				
	SGA ^5^	0.64 (0.45–0.90) *	0.65 (0.45–0.94) *	1.24 (0.80–1.93)	1.22 (0.76–1.93)
	AGA ^6^	1	1	1	1
	LGA ^7^	0.87 (0.74–1.02)	0.88 (0.74–1.05)	1.05 (0.74–1.47)	0.96 (0.67–1.37)
FEEDING-RELATED FACTORS	**Any breastfeeding:**				
	no	0.12 (0.07–0.20) ***	0.25 (0.15–0.44) ***	0.36 (0.12–1.41)	0.36 (0.15–0.65) ***
	yes	1	1	1	1
	**Infant formula use at maternity ward:**				
	no	1	1	1	1
	otherwise	0.65 (0.57–0.74) ***	0.88 (0.77–1.02)	0.71 (0.55–0.90)	0.89 (0.64–1.22)
	**Infant formula use after discharge:**				
	no	1	1	1	1
	yes	0.36 (0.31–0.42) ***	0.45 (0.38–0.54) ***	0.53 (0.40–0.70) ***	0.37 (0.25–0.55) ***

^1^ OR—univariate model; ^2^ CI—confidence intervals; ^3^ aOR—multivariate model; ^4^ Average monthly income per capita categories depends on country: 1st category <500 PLN (Poland)/<1000 EUR (Austria); 2nd category 500–1000 PLN/1000–1500 EUR; 3rd category 1001–2000 PLN/1501–2000 EUR; 4th category 2001–2500 PLN/2001–3000 EUR; 5th category 2501–3000 PLN/3001–5000 EUR; 6th category ≥3001 PLN/≥5001 EUR; ^5^ SG—small to gestational age; ^6^ AGA—appropriate to gestational age; ^7^ LGA—large to gestational age; * *p*-value ≤ 0.05; ** *p*-value ≤ 0.01; *** *p*-value ≤ 0.001.

## Supplementary Materials

The following are available online at https://www.mdpi.com/1660-4601/16/20/3799/s1, Table S1: Study group sociodemographic characteristics according to the age of introducing complementary feeding into infant diet; Table S2: Children’s characteristics, including birth parameters and feeding methods; Table S3: Univariate and multivariate regression analysis models of factors influencing the complementary feeding introduction before 4 months according to duration of pregnancy.
