# Supplementary material for: Factors Influencing the Age of Complementary Feeding—A Cross-Sectional Study from Two European Countries
Source: Int J Environ Res Public Health. 2019 Oct 9;16(20):3799. doi: 10.3390/ijerph16203799 (PMC6843416; doi:10.3390/ijerph16203799)
Supplement: Supplementary file 1 [file ijerph-16-03799-s001.pdf]

International Journal of
Environmental Research and Public Health

MDPI

Article

Factors influencing the age of complementary feeding – a cross-sectional study from two European countries

Monika A. Zielinska [1] , Petra Rust [2,*], Daria Masztalerz-Kozubek[1], Jacqueline Bichler[2] and Jadwiga Hamułka [1]

Table S1. Study group socio-demographic characteristics according to the age of introducing complementary feeding into infant diet.

Variable	Poland (n= 4065) % (n)				Austria (n= 1750) % (n)			
	< 4 mo	4-6 mo	> 6 mo	*p*-value	< 4 mo	4-6 mo	> 6 mo	*p*-value
**Maternal age:**								
- < 25 years	9.2 (9)	5.5 (134)	3.2 (48)		6.6 (5)	4.2 (55)	5.6 (20)	
- 25-29 years	34.7(34)	34.9 (860)	28.3 (426)	≤ 0.001	32.9 (25)	26.2 (345)	24.4 (87)	0.298
- 30-34 years	39.8 (39)	45.2 (1111)	52.3 (788)		26.3 (20)	40.5 (534)	38.7 (138)	
- 35-39 years	14.3 (14)	13.1 (322)	14.4 (218)		30.2 (23)	24.5 (322)	25.5 (91)	
- ≥ 40 years	2.0 (2)	1.3 (33)	1.8 (27)		4.0 (3)	4.6 (61)	5.8 (21)	
**Paternal age:**								
- < 25 years	5.1 (5)	1.7 (43)	0.7 (11)		4.0 (3)	2.0 (27)	1.4 (5)	
- 25-29 years	23.5 (23)	21.4 (526)	18.2 (274)	≤ 0.001	15.8 (12)	14.3 (188)	14.0 (50)	0.519
- 30-34 years	42.9 (42)	48.8 (1200)	49.3 (743)		36.8 (28)	34.9 (459)	31.1 (111)	
- 35-39 years	20.4 (20)	21.1 (518)	25.6 (386)		25.0 (19)	30.5 (402)	30.8 (110)	
- ≥ 40 years	8.1 (8)	7.0 (173)	6.2 (93)		18.4 (14)	18.3 (241)	22.7 (81)	
**Maternal education:**								
- primary and vocational	6.1 (6)	1.0 (25)	0.5 (7)	≤ 0.001	34.2 (26)	26.2 (345)	24.4 (87)	0.048
- high school	19.4 (19)	12.8 (316)	9.4 (142)		7.9 (6)	3.1 (41)	4.5 (16)	
- University	74.5 (73)	86.2 (2119)	90.1 (1358)		57.9 (44)	70.7 (931)	71.1 (254)	
**Paternal education:**								
- primary and vocational	17.4 (17)	10.5 (258)	6.5 (98)	≤ 0.001	48.7 (37)	45.0 (593)	44.0 (157)	0.531
- high school	25.5 (25)	24.9 (613)	20.7 (312)		10.5 (8)	6.4 (84)	6.7 (24)	
- University	57.1 (56)	64.6 (1589)	72.8 (1097)		40.8 (31)	48.6 (640)	49.3 (176)	
**Household size:**								
- 2	3.1 (3)	1.8 (45)	2.2 (33)		3.9 (3)	2.1 (28)	2.2 (8)	
- 3	65.3 (64)	62.6 (1539)	61.6 (928)		53.9 (41)	47.5 (626)	44.0 (157)	
- 4	24.5 (24)	28.2 (694)	28.6 (431)	0.937	31.6 (24)	38.1 (502)	37.8 (135)	0.521
- 5	6.1 (6)	5.4 (132)	5.8 (87)		6.6 (5)	8.5 (112)	11.5 (41)	
- ≥ 6	1.0 (1)	2.0 (50)	1.9 (28)		3.9 (3)	3.7 (49)	4.5 (16)	
**Number of children in household:**								
- 1	69.4(68)	68.4 (1682)	66.9 (1008)		55.3 (42)	49.4 (651)	45.4 (162)	
- 2	24.5 (24)	27.8 (683)	28.0 (422)	0.344	28.9 (22)	37.4 (492)	35.6 (127)	0.044
- ≥ 3	6.1 (6)	3.8 (95)	5.1 (77)		15.8 (12)	13.2 (174)	19.0 (68)	
**Living area:**								
- rural	17.4 (17)	18.5 (454)	15.5 (234)	0.061	59.2 (45)	57.6 (758)	51.3 (183)	0.091
- urban	82.6 (81)	81.5 (2006)	84.5 (1273)		40.8 (31)	42.4 (559)	48.7 (174)	
**Living macroeconomic region (EU-28 GDP):**								
- 47-50%	25.5 (25)	15.2 (375)	10.9 (164)		-	-	-	
- 51-100%	50.0 (49)	61.5 (1513)	65.6 (988)		6.6 (5)	5.1 (68)	6.4 (23)	
- 101-110%	24.5 (24)	23.3 (572)	23.5 (355)	≤ 0.001	38.2 (29)	41.3 (544)	45.9 (164)	0.080
- 111-130%	-	-	-		25.0 (19)	26.9 (354)	24.1 (86)	
- 131-150%	-	-	-		7.9 (6)	11.5 (151)	6.2 (22)	
- >150%	-	-	-		23.3 (17)	15.2 (200)	17.4 (62)	

**Average monthly income per capita[1]:**								
- 1st category	2.0 (2)	1.6 (39)	1.2 (19)	0.235	9.2 (7)	8.3 (109)	9.2 (33)	0.861
- 2nd category	22.4 (22)	14.5 (357)	13.8 (210)		23.7 (18)	21.0 (276)	19.6 (70)	
- 3rd category	23.5 (23)	33.0 (812)	32.8 (499)		27.6 (21)	32.0 (422)	30.0 (107)	
- 4th category	17.4 (17)	17.0 (419)	17.8 (271)		21.1 (16)	26.5 (349)	27.2 (97)	
- 5th category	7.1 (7)	12.4 (306)	12.3 (187)		15.8 (12)	10.0 (132)	11.8 (42)	
- 6th category	27.6 (27)	21.4 (527)	22.0 (335)		2.6 (2)	2.2 (29)	2.2 (8)	

[1] Average monthly income per capita categories are depend on country: 1st category < 500 PLN (Poland) or < 1000 EUR (Austria); 2nd category 500 – 1000 PLN or 1000 - 1500 EUR; 3rd category 1001 – 2000 PLN / 1501 – 2000 EUR; 4th category 2001 – 2500 PLN / 2001 – 3000 EUR; 5th category 2501 – 3000 PLN / 3001 – 5000 EUR; 6th category ≥ 3001 PLN / ≥ 5001 EUR.

Table S2. Children's characteristics including birth parameters and feeding methods.

Variable	Poland (*n*= 4065) Mean ± SD [1] Min - Max % (n)	Austria (*n*= 1750) Mean ± SD Min - Max % (n)	*p*-value
**Infant age:**	21.8 ± 7.2<br>12.0 – 36.0	23.9 ± 7.7<br>12.0 – 36.0	≤ 0.001
**Infant gender:**			
- female	47.7 (1938)	50.1 (877)	0.088
- male	52.3 (2127)	49.9 (873)	
**Pregnancy duration:**			
- <37 weeks	6.4 (260)	6.3 (110)	0.436
- 37-42 weeks	93.1 (3784)	92.9 (1626)	
- >42 weeks	0.5 (21)	0.8 (14)	
**Birthweight to gestational age categories:**			
- SGA[2]	4.1 (167)	6.7 (118)	≤ 0.001
- AGA[3]	76.8 (3123)	80.1 (1401)	
- LGA[4]	19.1 (775)	13.2 (231)	
**Any breastfeeding:**			
- no	5.3 (215)	8.6 (150)	≤ 0.001
- yes	94.7 (3850)	91.4 (1600)	
**Infant formula use at maternity ward:**			
- no	40.5 (1647)	61.4 (1074)	≤ 0.001
- don't know	3.9 (159)	0.5 (9)	
- yes	55.6 (2259)	38.1 (667)	
**Infant formula use after discharge:**			
- no	68.0 (2765)	68.0 (1190)	0.988
- yes	32.0 (1300)	32.0 (560)	
**Age of introduction of complementary feeding:**			
- < 4 months	2.4 (98)	4.3 (76)	≤ 0.001
- 4-6 months	60.5 (2460)	75.3 (1317)	
- > 6 months	37.1 (1507)	20.4 (357)	
**Complementary feeding method:**			
- TSF [5]	28.1 (1144)	36.5 (639)	≤ 0.001
- partially BLW	54.6 (2218)	49.4 (865)	
- BLW [6]	17.3 (703)	14.1 (246)	

[1] SD – standard deviation; [2] SGA – small to gestational age; [3] AGA – appropriate to gestational age; [4] LGA – large to gestational age; [5] TSF—traditional spoonfeeding; [6] BLW—baby-led weaning.

Table S3. Univariate and multivariate regression analysis models of factors influencing the complementary feeding introduction before 4 months according to duration of pregnancy.

	Variable	Preterm infants (*n*= 370)		Term infants (*n*= 5445)	
		**OR [1] (95% CI [2])**	**aOR [3] (95% CI)**	**OR (95% CI)**	**aOR (95% CI)**
SOCIO-DEMOGRAPHIC FACTORS	**Maternal age:**				
	- < 25 years	1.99 (0.73 – 5.46)	1.76 (0.49 – 6.29)	6.61 (2.33 – 18.73)***	3.44 (1.11 – 10.61)*
	- 25-29 years	1.31 (0.78 – 2.21)	1.78 (0.92 – 3.45)	3.47 (1.59 – 7.60)**	2.82 (1.26 – 6.31)*
	- 30-34 years	1	1	1	1
	- 35-39 years	1.01 (0.56 – 1.83)	1.08 (0.51 – 2.25)	4.24 (1.83 – 9.83)***	3.45 (1.45 – 8.20)**
	- ≥ 40 years	0.90 (0.27 – 2.99)	0.65 (0.15 – 2.84)	2.07 (0.26 – 16.46)	1.09 (0.13 – 9.04)
	**Maternal education:**				
	- primary and vocational	3.77 (1.63 – 8.70)**	2.27 (0.78 – 6.56)	5.73 (3.13 – 10.52)***	3.01 (1.41 – 6.45)**
	- high school	3.14 (1.56 – 6.34)**	3.84 (1.58 – 9.33)**	1.53 (0.59 – 3.98)	1.22 (0.44 – 3.37)
	- University	1	1	1	1
	**Number of children in household:**				
	- 1	1.02 (0.61 – 1.70)	0.70 (0.37 – 1.32)	0.88 (0.48 – 1.60)	0.93 (0.49 – 1.77)
	- 2	1	1	1	1
	- ≥ 3	1.76 (0.77 – 4.05)	1.62 (0.57 – 4.56)	0.99 (0.33 – 2.94)	0.79 (0.26 – 2.43)
	**Country:**				
	- Austria	1.62 (1.02 – 2.57)*	2.59 (1.28 – 5.27)**	3.65 (2.07 – 6.42)***	2.13 (1.01 – 4.51)*
	- Poland	1	1	1	1
	**Living area:**				
	- rural	0.99 (0.63 – 1.56)	0.77 (0.41 – 1.43)	1.61 (0.91 – 2.83)	0.84 (0.44 – 1.59)
	- urban	1	1	1	1
	**Average monthly income per capita[4]:**				
	- 1st category	0.93 (0.28 – 3.10)	0.27 (0.06 – 1.17)	2.88 (0.86 – 9.65)	1.98 (0.56 – 6.93)
	- 2nd category	1.42 (0.70 – 2.89)	0.93 (0.37 – 2.32)	2.02 (0.83 – 4.89)	1.86 (0.74 – 4.69)
	- 3rd category	0.55 (0.29 – 1.06)	0.58 (0.26 – 1.31)	1.25 (0.53 – 2.93)	1.43 (0.59 – 3.44)
	- 4th category	1	1	1	1
	- 5th category	0.55 (0.24 – 1.24)	0.73 (0.28 – 1.90)	1.52 (0.55 – 4.21)	2.01 (0.70 – 5.76)
	- 6th category	1.04 (0.52 – 2.09)	1.71 (0.71 – 4.09)	0.48 (0.13 – 1.82)	0.97 (0.24 – 3.92)
PREGNANCY-RELATED FACTORS	**Infant gender:**				
	- female	1	1	1	1
	- male	0.80 (0.52 – 1.24)	0.79 (0.46 – 1.37)	1.24 (0.71 – 2.17)	1.35 (0.76 – 2.39)
	**Pregnancy duration [Hbd[5]]:**	0.69 (0.62 – 0.77)***	0.67 (0.58 – 0.76)***	1.15 (0.92 – 1.41)	1.12 (0.92 – 1.37)
	**Birthweight to gestational age categories:**				
	- SGA[6]	1.20 (0.55 – 2.61)	1.12 (0.44 – 2.81)	2.23 (0.87 – 5.71)	1.76 (0.66 – 4.74)
	- AGA[7]	1	1	1	1
	- LGA[8]	1.40 (0.81 – 2.42)	1.68 (0.83 – 3.39)	0.96 (0.44 – 2.06)	1.08 (0.49 – 2.38)
FEEDING-RELATED FACTORS	**Any breastfeeding:**				
	- no	4.83 (2.38 – 9.78)***	0.27 (0.36 – 0.87)**	2.11 (0.90 – 4.99)	0.70 (0.26 – 1.86)
	- yes	1	1	1	1
	**Infant formula use at maternity ward:**				
	- no	1	1	1	1
	- otherwise	1.84 (1.13 – 2.99)*	1.75 (0.92 – 3.35)	0.88 (0.51 – 1.52)	0.39 (0.20 – 0.77)**
	**Infant formula use after discharge:**				
	- no	1	1	1	1
	- yes	2.73 (1.75 – 4.26)***	2.01 (1.11 – 3.63)*	5.40 (2.95 – 9.90)***	7.81 (3.92 – 15.54)***

[1] OR – univariate model; [2] CI – confidence intervals; [3] aOR – multivariate model; [4] Average monthly income per capita categories are depend on country: 1st category < 500 PLN (Poland) or < 1000 EUR (Austria); 2nd category 500 – 1000 PLN or 1000 - 1500 EUR; 3rd category 1001 – 2000 PLN / 1501 – 2000 EUR; 4th category 2001 – 2500 PLN / 2001 – 3000 EUR; 5th category 2501 – 3000 PLN / 3001 – 5000 EUR; 6th category ≥ 3001 PLN / ≥ 5001 EUR; [5] Hbd – hebdomas (week); [6] SGA – small to gestational age; [7] AGA – appropriate to gestational age; [8] LGA – large to gestational age; * p-value ≤ 0.05; ** p-value ≤ 0.01; *** p-value ≤ 0.001.
